# Extracellular ATP activates NFAT-dependent gene expression in neuronal PC12 cells via P2X receptors

**DOI:** 10.1186/1471-2202-12-90

**Published:** 2011-09-23

**Authors:** Prabin Prasai, Georgios C Stefos, Walter Becker

**Affiliations:** 1 Institute of Pharmacology and Toxicology, Medical Faculty of the RWTH Aachen University, Wendlingweg 2, 52074 Aachen, Germany

## Abstract

**Background:**

Treatment of neuronal PC12 cells with ATP induces depolarisation and increases intracellular calcium levels via purinergic receptors. In many cell types, sustained elevation of intracellular calcium levels cause changes in gene expression via activation of the transcription factor NFAT (nuclear factor of activated T cells). We have therefore characterised the signalling pathway by which ATP regulates NFAT-dependent gene expression in PC12 cells.

**Results:**

The activation of NFAT transcriptional activity by extracellular ATP was characterised with the help of reporter gene assays. Treatment of PC12 cells with ATP elicited a dose-dependent increase in luciferase activity (EC_50 _= 78 μM). UTP, 4-benzoylbenzoyl ATP and α,β-methylene ATP did not mimic the effect of ATP, which was abolished by treatment with the P2X receptor antagonist pyridoxal-phosphate-6-azophenyl-2',4'-disulfonate (PPADS). This pharmacological characterisation provides evidence for a critical role of ionotropic P2X receptors. Blockade of L-type voltage-dependent calcium channels by nifedipine reduced the response of NFAT to ATP, indicating that a depolarisation-mediated calcium influx was required for maximal NFAT activation. Inhibition of store-operated calcium entry by the pyrazole derivative BTP2 also diminished ATP-dependent NFAT activation. Furthermore, ATP-induced NFAT activation was associated with the activation of the mitogen-activated protein kinases ERK1/2. Finally, treatment with ATP increased the levels of the NFAT target transcripts, RCAN1-4 (regulator of calcineurin) and BDNF (brain derived neurotrophic factor).

**Conclusion:**

The present data show that ATP induces NFAT-dependent changes in gene expression in PC12 cells by acting on P2X receptors. Maximal NFAT activation depends on both depolarisation-induced calcium influx and store-operated calcium entry and requires the activity of the protein phosphatase calcineurin and the mitogen-activated protein kinase cascade.

## Background

Purinergic signalling plays a significant role in neurotransmission and neuromodulation in many regions of the brain as well as in the spinal cord and peripheral neurons [1,2]. Among purinergic agonists, extracellular ATP is a potent signalling molecule abundantly present in the central nervous system. ATP is secreted from many neurons as a cotransmitter by vesicular exocytotic release, but also leaks from injured or dying cells [3]. In addition, many cell types, including glial cells, can also release ATP in response to stimuli such as hypoxia or certain agonists independently of cell damage and thereby modulate the function of adjacent neurons.

Once released into the extracellular space, ATP acts on specific receptors that belong to two main subclasses: ligand-gated P2X cation-selective channels and G protein-coupled P2Y receptors [4]. Both receptor classes evoke calcium responses. P2X receptors can induce depolarisation-induced calcium entry and are permeable to Ca^2+^, whereas most P2Y receptors couple to phospholipase Cβ isoforms, which leads to the release of Ca^2+ ^from internal stores. Purinergic effects mediated by Ca^2+ ^signalling include presynaptic neurotransmitter release, hormone secretion, calcium wave propagation between astrocytes and activation of primary nociceptive neurons [3,5-7].

Although purinergic receptors are abundant in the nervous system and have been extensively characterised with respect to their short-term effects on neuronal function, less is known about the long-term effects of their activation in neurons. There is evidence that extracellular nucleotides affect neuronal differentiation and survival, but the signalling pathways that mediate these effects are largely unexplored [8]. Specifically, the increase in intracellular Ca^2+ ^concentrations after activation of purinergic receptors is expected to influence gene expression. The calcineurin-NFAT (nuclear factor of activated T-cells) pathway is a major mediator of Ca^2+ ^effects on gene expression in neuronal cells and plays a key role in neuronal development and function [9-11]. Surprisingly, the effects of purinergic receptors on NFAT signalling and NFAT-dependent gene expression have not yet been studied in neuronal cells.

The rat pheochromocytoma cell line PC12 is a well-characterised model system for purinergic effects. PC12 cells express P2X and P2Y receptors and show increases in intracellular Ca^2+ ^concentration upon stimulation with extracellular ATP [12-16]. Extracellular ATP stimulates catecholamine release from PC12 cells, enhances their sensitivity to nerve growth factor, promotes neurite outgrowth and regulates cytoskeleton remodelling [13,15,17,18]. Moreover, PC12 cells express the components of the calcineurin-NFAT pathway and have been used to characterise NFAT-dependent changes in gene expression [19-21].

Here we have tested the hypothesis that extracellular ATP can modulate gene expression in neuronal cells via the calcineurin-NFAT pathway. We show that ATP stimulates NFAT transcriptional activity through the activation of P2X receptors, causes the activation of ERK1/2 kinases and induces the expression of an NFAT target gene in PC12 cells. These results suggest that extracellular ATP can act on neuronal cells by inducing NFAT-dependent changes in gene expression.

## Results

### Extracellular ATP induces NFAT-dependent reporter gene activity in PC12 cells

**Figure 1:**
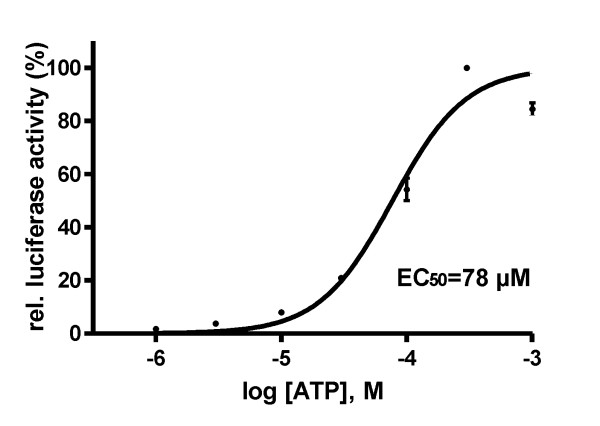
**Concentration-dependent induction of NFAT-driven reporter gene expression by extracellular ATP**. PC12-NFAT-Luc cells were treated with varying concentrations of ATP for 3 h before lysis. Luciferase activities were normalised to the maximum induction obtained with 300 μM ATP. The graph presents means ± SD of n = 3 independent experiments. Nonlinear curve-fitting yielded EC_50 _= 78 μM ATP (95% confidence interval 62-95 μM ATP).

To study the effect of extracellular ATP on the activation of NFAT in neuronal cells, we generated a stable PC12 subclone expressing luciferase under the control of a NFAT-driven promoter (PC12-NFAT-Luc). Treatment of PC12-NFAT-Luc cells with ATP strongly induced luciferase activity, with a maximal response at 300 μM ATP (58 ± 12-fold increase, mean and S.D. of 3 independent experiments) (Figure 1). Significant stimulation of NFAT activation was detected at a concentration as low as 1 μM ATP (2.93 ± 0.23-fold induction, mean ± S.D.). The half-maximal effect was produced at a concentration of EC_50 _= 78 μM ATP. It is important to note that the actual concentration of ATP is not constant during the incubation time of 3 h because PC12 cells express multiple ecto-ATPases [22]. Under the conditions of this experiment, the half life of ATP was ~ 40 min (data not shown). No obvious toxicity was observed in the trypan blue uptake test after treatment of the cells with 300 μM ATP for 3 h.

### Pharmacological characterisation of purinergic receptors that mediate NFAT activation in PC12 cells

**Figure 2:**
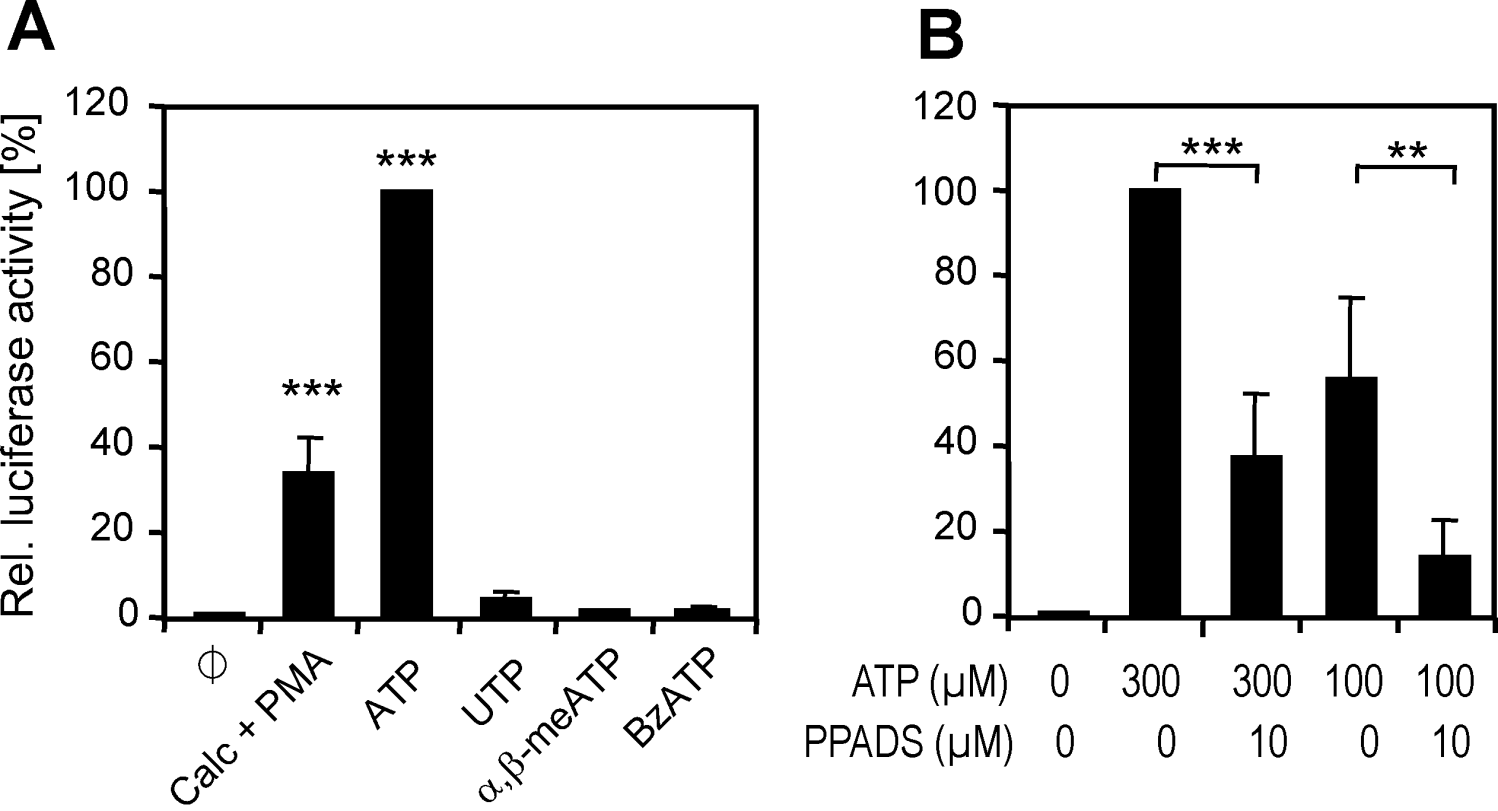
**Pharmacological characterisation of purinergic receptors that mediate NFAT activation**. **A**, Effects of purinergic agonists. Cells were treated with the indicated compounds at the following final concentrations: 300 μM ATP, 300 μM UTP, 30 μM α,β-MeATP, 30 μM BzATP or 10 μM calcimycin + 1 μM PMA. Ø, untreated control cells. **B**, Effect of the P2X antagonist PPADS. PC12-pNFAT-Luc cells were treated with ATP and PPADS as indicated before the cells were lysed, and luciferase activity was measured. Luciferase activities are expressed as the fold stimulation by the treatment relative to that in cells treated with 300 μM ATP alone. Data are shown as mean ± SD of 4 independent experiments except for Calc+PMA (n = 3). Statistical significance is indicated for differences vs. control (panel A) or vs. 300 μM ATP (panel B) **, p < 0.01; ***, p < 0.001.

We aimed to characterise the purinergic receptor responsible for the stimulatory effect of ATP on NFAT with different agonists and antagonists. For comparison, we used the calcium ionophore calcimycin (A23187) in combination with the PKC activator, PMA. This treatment serves as a positive control to activate NFAT in a receptor-independent manner [23]. As shown in Figure 2, maximal induction of NFAT-dependent promoter activity by ATP exceeded that elicited by calcimycin/PMA. In contrast, UTP, which is an agonist of some P2Y receptor subtypes, only marginally stimulated reporter gene activity. The ATP derivatives α,β-meATP, which acts as an agonist on receptors containing P2X1 or P2X3 subunits [24], and BzATP, which can activate several P2X subtypes (P2X1-4; [24]) and human P2Y11, had minimal effects on NFAT.

To further examine the receptors responsible for NFAT activation in PC12 cells, we used the P2X receptor antagonist PPADS (Figure 2B). Treatment of the PC12-NFAT-Luc cells with 10 μM PPADS strongly suppressed the induction of luciferase activity by ATP, suggesting that at least one of the PPADS-sensitive P2X subunits (P2X1, P2X2, P2X3 or P2X5; [24]) is involved in NFAT activation.

### Expression analysis of P2X receptor subunits and NFAT isoforms in PC12 cells

**Figure 3:**
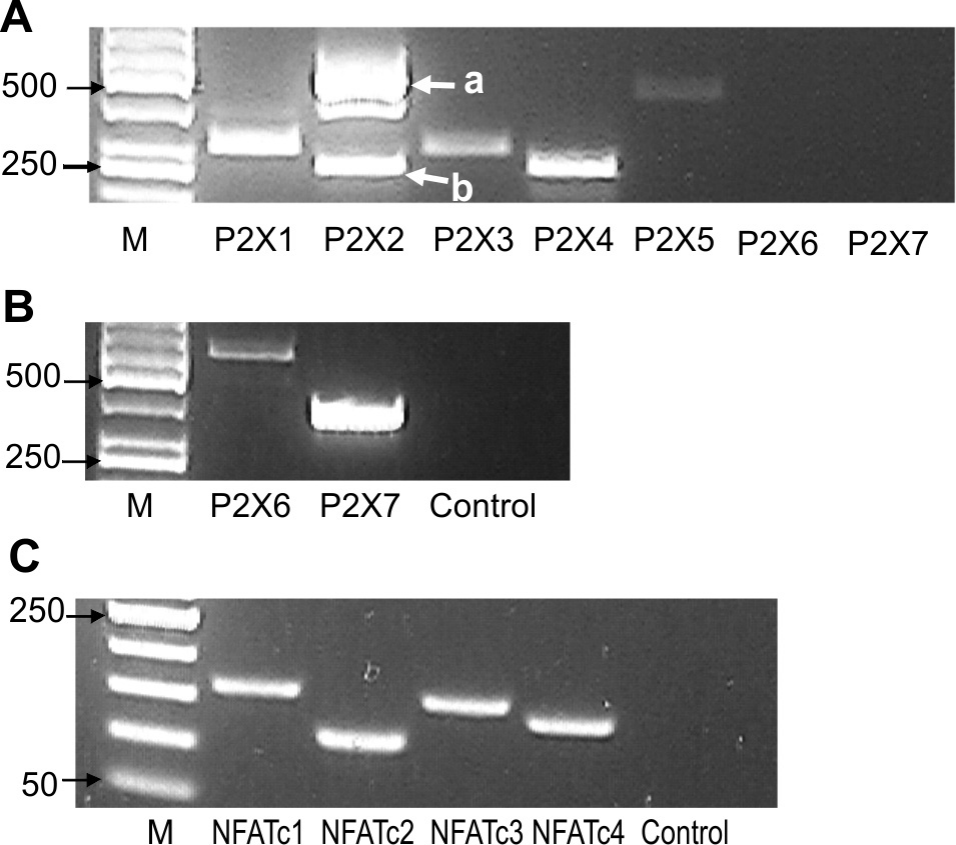
**Expression analysis of NFAT isoforms and P2X receptor subunits**. RT-PCR expression analysis of undifferentiated PC12-NFAT-Luc cells with PCR primers specific for the indicated P2X receptor (A) or NFAT isoforms (C). Panel B shows positive controls for the P2X receptors that were not detected in undifferentiated PC12-NFAT-Luc cells. For the amplification of P2X6, cDNA from NGF-treated PC12-NFAT-Luc cells used as template. P2X7 was amplified from a plasmid containing mouse P2X7 cDNA. Negative control reactions without DNA template (*control*) are presented for the primer sets for P2X6 and P2X7 (panel B) and the combined primers sets for the 4 NFAT isoforms (panel C). Migration of size markers (M) is indicated in bp.

The presence of the mRNA for the seven P2X receptor subtypes was analysed by RT-PCR. As shown in Figure 3A, bands of the expected size were detected for P2X1 and P2X3-5. A more complex pattern of bands was obtained with the P2X2-specific primers. Sequencing revealed that the two main bands corresponded to variants of P2X2 (P2X2a and P2X2b) that differ by an alternatively spliced region in the C-terminal domain [25]. Although P2X2 appears to be most strongly expressed among the P2X receptors, it must be noted that bands obtained by end-point PCR amplification of different target sequences cannot be quantitatively compared. Transcripts for P2X6 and P2X7 were below the detection level under our conditions (Figure 3A). Expression of all of the 4 Ca^2+^-responsive NFATc isoforms was readily shown by RT-PCR (Figure 3C).

### Mechanisms of cytosolic Ca^2+ ^increase

**Figure 4:**
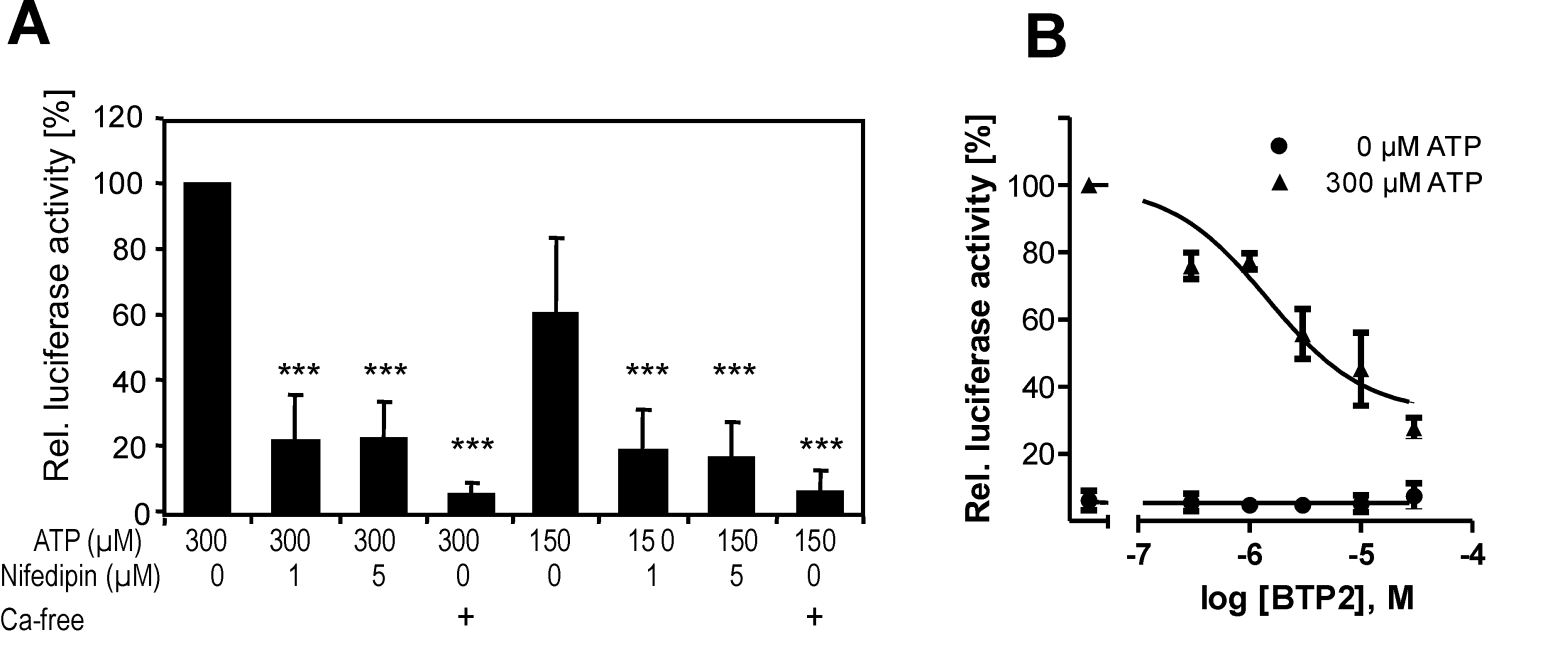
**Ca^2+ ^channel blockers inhibit ATP-induced NFAT activation**. **A**, Effect of nifedipine and dependence on extracellular calcium. PC12-NFAT-Luc cells were treated for 3 h with ATP and nifedipine as indicated or were stimulated with ATP in the absence of extracellular free Ca^2+^. Nominal Ca^2+^-free conditions were created using 2 mM EGTA. Statistical significance is indicated for differences vs. the control cells treated only with 300 μM ATP or 150 μM ATP ***, p < 0.001. **B**, Effect of BTP2. Cells were treated with varying concentrations of BTP2 during ATP stimulation. A simple inhibitor-response curve was tentatively fitted to the data, although more than one mechanism may contribute to the inhibitory effect of BTP2. The calculated IC_50 _is 1.5 μM (95% confidence interval 0.7-3.3 μM). Luciferase activities are expressed as the fold stimulation by the treatment relative to that in cells only treated with 300 μM ATP. Both graphs show means ± SD of 3 independent experiments.

Activation of NFAT depends on elevated Ca^2+ ^concentrations in the cytosol. Our tentative identification of a P2X receptor raises questions about the molecular mechanism of the Ca^2+ ^response. Depletion of extracellular Ca^2+ ^prevented the induction of luciferase activity (Figure 4A), supporting the notion that Ca^2+ ^influx from the extracellular space is required for the activation of NFAT by ATP. The Ca^2+ ^needed for activation of calcineurin could enter the cell directly through P2X cation channels and/or *via *voltage-gated Ca^2+ ^channels that open as a consequence of P2X-mediated membrane depolarisation.

To test the latter possibility, we studied the effect of the L-type calcium channel blocker, nifedipine, on the induction of luciferase by ATP at the optimal concentration of ATP in this assay (300 μM) as well as a suboptimal concentration of 150 μM ATP (Figure 4A). Nifedipine strongly reduced NFAT activation but did not completely prevent the effect of ATP, indicating that a major part of the NFAT response depends on Ca^2+ ^influx through L-type Ca^2+ ^channels.

Next we asked whether store-operated Ca^2+ ^entry (SOCE) contributed to the effect of ATP on NFAT activation. The pyrazole derivative BTP2 is a blocker of SOCE and inhibits NFAT effects in different cell types, including T lymphocytes and cardiomyocytes [26,27]. Treatment with BTP2 reduced NFAT activation in PC12 cells in a concentration-dependent manner (Figure 4B). Partial but significant inhibition was observed at submicromolar concentrations (0.3 μM), at which BTP2 is thought to specifically inhibit SOCE [28,29]. A maximal effect of 72% inhibition was observed at a concentration of 30 μM BTP2. It must be noted that the direct molecular target(s) of BTP2 are still not well defined [29], and the unsteady slope of the concentration-response curve might suggest that there is more than one target affected by BTP2. Taken together, these results suggest that the maximal activation of NFAT by extracellular ATP in PC12 cells requires the influx of extracellular Ca^2+ ^ions both *via *voltage-dependent calcium channels and a BTP2-sensitive mechanism.

### Pathway for the ATP activation of NFAT

**Figure 5:**
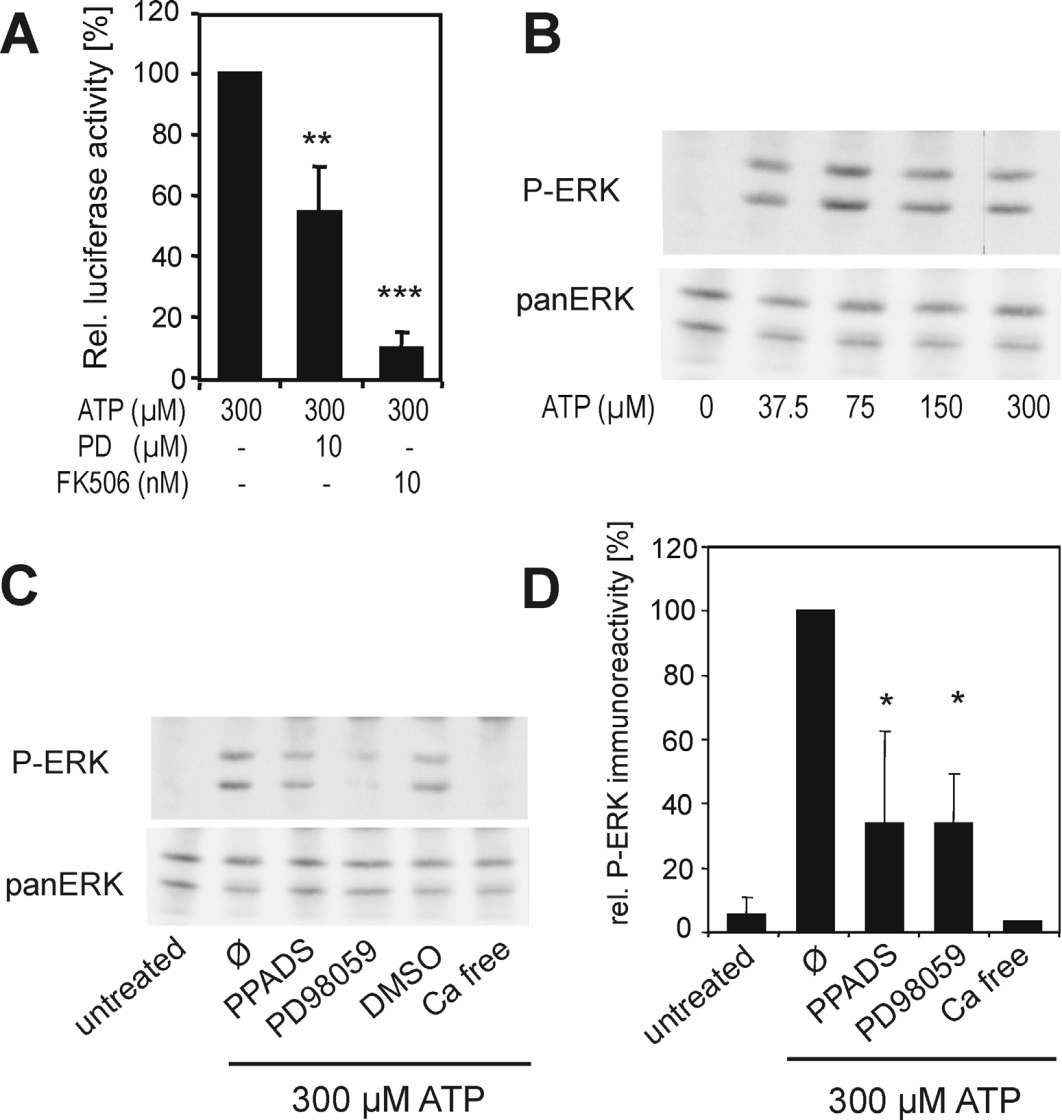
**ATP-induced MEK-ERK1/2 signalling contributes to NFAT activation**. **A**, Effect of FK506 and PD98059 on ATP-induced NFAT activity. Cells were treated with ATP and the inhibitors as indicated. **B **and **C**, Activation of ERK1/2 by extracellular ATP. PC12 cells were stimulated with varying concentrations of ATP as indicated for 10 min before cells were lysed (**B**). Activation of ERK1 and ERK2 was detected by Western blot analysis with antibodies specific for the phosphorylated forms of ERK1 and ERK2 (P-ERK) and total ERK1/2 (panERK). In **C **and **D**, cells were pretreated for 30 min with either PPADS (10 μM), PD98059 (10 μM) or solvent (DMSO) or were not pretreated (Ø) before stimulation with ATP, or were kept under Ca^2+^-free conditions. P-ERK band intensities were quantified and normalised to the corresponding panERK signal. The graph in panel **D **presents the results of three independent experiments (means ± SD). Treatment with Ca^2+^-free medium was performed only twice, and here the mean of the two values is shown. Statistical significance of the results in A and D is indicated for differences vs. the control cells treated only with 300 μM ATP (*, p < 0.05; **, p < 0.01. ***, p < 0.001).

We used specific inhibitors of protein phosphatase 2B/calcineurin (FK506, also known as tacrolimus) and protein kinase MEK1 (PD98059) to examine the intracellular pathways involved in NFAT activation. FK506 suppressed ATP-induced luciferase activity, confirming that the reporter gene expression indeed depended on the calcineurin-NFAT pathway (Figure 5A). Interestingly, the MEK inhibitor PD98059 also reduced reporter gene activity, suggesting that activation of the MEK/ERK1/2 cascade was required for maximal induction of NFAT transcriptional activity. To verify that extracellular ATP can activate the MEK/ERK pathway, PC12 cells were treated with varying concentrations of ATP, and activation of ERK1/2 was monitored by Western blot analysis with an activation-state specific antibody (Figure 5B). Phosphorylation of ERK1/2 was well detectable after treatment with 37.5 μM of ATP. This response was fully dependent on extracellular Ca^2+ ^and was partially inhibited by PPADS (Figure 5C), suggesting that ERK1/2 phosphorylation required the activation of P2X receptors.

### Effect of extracellular ATP on NFAT target genes

**Figure 6:**
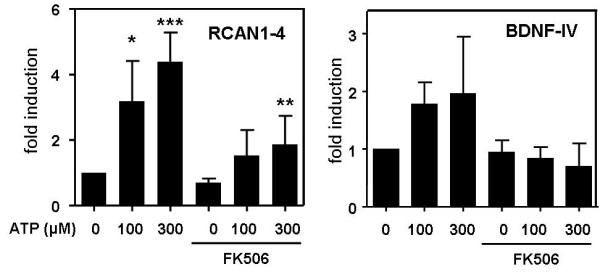
**Effect of ATP treatment on mRNA levels of NFAT target genes**. PC12-NFAT-Luc cells were treated with varying concentrations of ATP as indicated and FK506 (10 nM) for 3 hours. RCAN1-4 mRNA and exon VI-containing BDNF mRNA were measured by real-time PCR quantification, and the results are shown as the fold induction relative to untreated cells. The graph shows the means ± SD of 3 independent experiments. Statistical significance is indicated for differences of ATP-treated cells vs. the control cells and for FK506 + 300 μM ATP-treated cells vs. cells treated only with 300 μM ATP (*, p < 0.05; **, p < 0.01. ***, p < 0.001).

Finally, we aimed to confirm that extracellular ATP regulates the expression of endogenous NFAT target genes in PC12 cells. Firstly, we examined the effect of ATP on mRNA levels of RCAN1-4 (also known as DSCR1 or calcipressin [30]), which is a target transcript of NFAT in various cell types, including neurons [20,31]. As shown in Figure 6A, transcript levels of RCAN1-4 were significantly induced by treatment with ATP, indicating that the activation of NFAT by purinergic receptors elicits transcriptional changes in PC12 cells. The upregulation of RCAN1-4 mRNA depended on the activation of the calcineurin-NFAT pathway, as confirmed by the inhibitory effect of FK506. As a second NFAT target transcript, we analysed the levels of the exon IV-containing transcripts of the *Bdnf *gene. Transcripts of the *Bdnf *gene can initiate with either of at least 8 different 5'-exons [32], of which the *Bdnf *exon IV-containing transcripts are selectively induced by the influx of extracellular calcium [33], and promoter IV is known to contain a NFAT response element [34]. Similar to RCAN1-4, we observed an increase of the *Bdnf*-IV transcripts in ATP stimulated cells that was blocked by treatment with FK506 (Figure 6B). However, these changes did not reach the level of statistical significance.

## Discussion

Our results show that the Ca^2+ ^response elicited by extracellular ATP in neuronal cells translates into changes in gene expression that are mediated by the transcription factor NFAT. In the PC12 cells that we used as a model system, NFAT activation by ATP required the influx of Ca^2+ ^from the extracellular space and depended on the activation of P2X receptors and the function of L-type voltage-dependent Ca^2+ ^channels. The calcineurin-NFAT pathway plays an important role in neuronal development, and our results support the assumption that purinergic receptors transmit some of their trophic effects by this mechanism [2].

**Figure 7:**
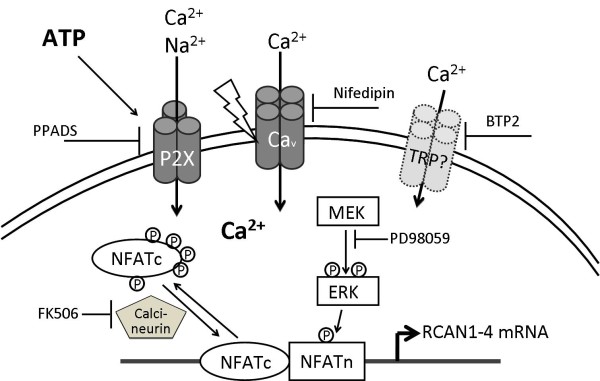
**Model of the proposed pathway by which extracellular ATP activates NFAT in PC12 cells**. The results demonstrate the requirement of Ca^2+ ^influx from the extracellular space and the contribution of both calcineurin-NFATc and MEK-ERK1/2 pathways. The scheme also illustrates the inhibitors used for pharmacological characterisation. BTP2 is a blocker of SOCE and inhibits TRPC channels, but its direct molecular target in PC12 cells is not clear.

Based on the results of the present study, we propose the following working model for the signalling events that result in NFAT activation (Figure 7). Firstly, the requirement of extracellular Ca^2+ ^and the inhibitory effect of nifedipine clearly indicate that the activation of ionotropic P2X receptors, as opposed to the metabotropic P2Y or P1 receptors, is essential for NFAT activation. Our result is in accordance with previous studies of ATP-induced Ca^2+ ^influx in PC12 cells [13,35], which showed that Na^+ ^influx through P2X2 receptors can cause sufficient membrane depolarisation to activate L-type voltage-gated Ca^2+ ^channels. The fact that even 5 μM of nifedipine incompletely blocked NFAT activation suggests that other mechanisms, such as direct Ca^2+ ^entry through the P2X receptor pore or a BTP2-sensitive channel (see below), contribute to the ATP-induced Ca^2+ ^response.

Secondly, the pharmacological characterisation of the purinergic receptor responsible for NFAT activation supports the hypothesis that a P2X receptor is critically involved. At a concentration of 10 μM, PPADS is an antagonist of homo- or heteromeric P2X complexes that contain P2X1, P2X2, P2X3 or P2X5 subunits but does not inhibit P2X4 and P2X6 [24,36]. Variable potencies have been reported for the inhibition of P2X7 by PPADS, with IC_50 _values ranging from ~100 nM to > 50 μM. However, we can exclude P2X7 here as the relevant receptor because 30 μM BzATP failed to induce luciferase expression despite the fact that rat P2X7 is highly responsive to BzATP in the low μM range [37]. In addition, P2X7 is a low affinity ATP receptor (EC_50 _> 100 μM [24]), whereas significant NFAT activation in PC12 cells was already detectable at low micromolar concentrations of ATP. The result that P2X7 does not account for NFAT activation by extracellular ATP in PC12 cells is important because P2X7 mediates NFAT activation in other cell types such as microglia and T cells [38-42]. Finally, the lack of an effect of 30 μM α,β -MeATP excludes the possibility that P2X1 or P2X3 induces the activation of NFAT in PC12 cells because these receptors are activated by submicromolar concentrations of this agonist [24]. Thus, P2X2 and P2X5 remain as the most likely candidates for activating receptors in this pathway.

Based on our data as well as published results, we favour the hypothesis that P2X2 is responsible for the major part of the ATP-induced Ca^2+^-influx and NFAT activation in our cell model for three reasons: i) the P2X2 receptor was originally cloned from PC12 cells [43] and appears to be the major P2X isoform in undifferentiated PC12 cells, whereas P2X5 was either reported to be undetectable [44] or found to be expressed at lower levels (Figure 3A, [15,44]), ii) calcium influx through P2X2 receptors results in the activation of the MAP kinase cascade [45], and iii) P2X2 differs from other P2X isoforms, including P2X5, in its slow desensitisation kinetics [46] and is thus capable of causing sufficient depolarisation of PC12 cells to induce Ca^2+ ^influx via L-type voltage-gated Ca^2+ ^channels [13,47]. The RT-PCR analysis of the PC12 clone used in the present study confirmed that the cells express both the slowly desensitising P2X2a isoform and the fast desensitising P2X2b splicing variants (Figure 3A).

Thirdly, we showed that, in addition to the dependence on calcineurin activity, maximal NFAT activation also depended on the activation of ERK1/2 (Figure 4). Similarly, MEK1-ERK1/2 signalling augments NFAT transcriptional activity in cultured neonatal cardiomyocytes [48,49]. As suggested by Sanna et al. [48], the MEK1-ERK1/2 pathway and the calcineurin-NFAT pathway may converge at the level of the transcription factors. Importantly, Ca^2+^-induced calcineurin activity alone is not always sufficient to activate NFAT-dependent transcriptional activity, but a second signal that activates the nuclear partner protein of NFAT (NFATn) may be required (Figure 6). AP1, which is a target of ERK1/2, is one of the nuclear partners of NFAT [50,51]. The simultaneous activation of calcineurin and the MEK-ERK1/2 pathway may explain the very strong ATP-induced NFAT activation that exceeds the effect elicited by the control treatment (PMA and calcimycin).

Finally, we have shown that BTP2 partially inhibited ATP-induced NFAT activation in PC12 cells (Figure 3B), indicating that a BTP2-sensitive Ca^2+ ^influx is necessary for the maximal response under the experimental conditions chosen (incubation time of 3 h). BTP2 is a selective inhibitor of capacitative Ca^2+ ^entry and does not affect Ca^2+ ^handling by mitochondria or endoplasmic reticulum or other Ca^2+ ^channels [29]. NFAT activation in lymphocytes strictly depends on store-operated Ca^2+ ^entry (SOCE)[29], but there exist conflicting data about the contribution of SOCE to Ca^2+ ^responses in PC12 cells [35,47,52]. Unfortunately, although several transient receptor potential (TRP) channels have been proposed as targets of BTP2 [28,53,54], the primary target of BTP2 is still a matter of debate, and the IC_50 _values obtained in different experimental systems vary significantly [29]. Therefore, the nature of the BTP2-sensitive mechanism in PC12 cells cannot be deduced from the present experiments, although a TRPC channel may contribute to Ca^2+ ^influx in PC12 cells.

We have found that extracellular ATP upregulates RCAN1-4 mRNA, which is known to be transcribed from an NFAT-regulated promoter [20,31]. *Rcan1 *encodes a negative feedback regulator of calcineurin and has multiple effects in neurons, including regulation of vesicle exocytosis, long-term potentiation and facilitation of apoptosis [55-58]. However, NFAT has many more functions in central and peripheral neurons, in particular as a transcription factor that transduces effects of neurotrophins and membrane depolarisation [9,10,20,59,60]. ATP is well known to change gene expression in neurons and has multiple effects on neurogenesis, neuronal differentiation and neuroprotection [8,61-63]. Based on the results obtained in neuronal PC12 cells as a model system, we propose that the calcineurin-NFAT pathway is a novel mechanism that mediates the trophic functions of extracellular ATP on neurons.

## Conclusions

Our analysis of ATP-induced NFAT activation in PC12 cells has identified a novel pathway through which extracellular ATP can regulate neuronal gene expression and thereby modulate the function and development of neurons. In contrast to immune cells and glia, where P2X7 mediates activation of NFAT, available evidence suggests that, in neurons, P2X2 accounts for this effect. P2X2 produces depolarisation-induced calcium influx through L-type Ca^2+ ^channels and the activation of MEK-ERK1/2 signalling, which are both required for maximal NFAT activation. We propose this pathway as a more general mechanism by which extracellular ATP can exert long-term effects on neuronal cells, which remains to be studied under physiological conditions.

## Methods

### Reagents

ATP, UTP, 3'-O-(4-benzoyl)benzoyl ATP (BzATP), nifedipine, α,β-MeATP (α,β-methylene-ATP) and PD98059 were obtained from Sigma (Taufkirchen, Germany). Phorbol 12-myristate 13-acetate (PMA), calcimycin (also known as A23187) and FK506 (Tacrolimus) were purchased from Calbiochem (San Diego, CA). Pyridoxal-phosphate-6-azophenyl-2',4'-disulfonate (PPADS) was obtained from Biotrend (Cologne, Germany) and BTP2 (a 3,5-bis(trifluoromethyl)pyrazole derivative, also called YM-58483) was obtained from Cayman Chemical (Ann Arbor, MI). Stock solutions of nifedipine, BTP2, FK506, PD98059 and PMA were prepared in DMSO. ATP, UTP and PPADS were dissolved in water, and calcimycin was dissolved in ethanol.

### Cell culture conditions

PC12 cells were grown in high glucose (4.5 g/L) Dulbecco's modified Eagles medium (DMEM) with L-glutamine and sodium pyruvate, supplemented with 5% foetal bovine serum (PAA Laboratories, Pasching, Austria), 10% horse serum (PAA) and 25 mM Hepes buffer (Sigma). Cells were grown to 60-80% confluency in 5% CO_2 _at 37°C in T75 filter flasks, and cultures were split every 48-72 h without trypsinisation. When indicated, nominal Ca^2+ ^-free conditions were created by adding EGTA to a final concentration of 2 mM. To assess cell viability after ATP treatment, the percentage of stained cells in the trypan blue uptake assay was calculated from hemacytometer counts of at least 300 cells per sample.

### Construction of PC12-NFAT-Luc cells

A PC12 subclone with a stably integrated NFAT-dependent luciferase reporter vector was generated in two steps. First, a Flp recombination target (FRT) site was integrated in the genome by transfecting PC12 cells with pFRT/LacZeo (Invitrogen, Carlsbad, CA) and selecting clones resistant to 100 μg/ml Zeocin. Second, the hygromycin resistance gene with a FRT site embedded in the 5' coding region was transferred from pcDNA5/FRT/TO (Invitrogen) into pNFAT-Luc (Stratagene, La Jolla, CA) to generate pNFAT-Luc/FRT. This plasmid was then integrated into the FRT-containing PC12 clone via Flp recombinase-mediated DNA recombination according to the manufacturer's instructions. Luciferase expression from pNFAT-Luc is under the control of four direct repeats of the NFAT binding sequence (GGAGGAAAAACTGTTTCATACAGAAGGCGT) from the IL-2 gene promoter.

### Reporter gene assays

For the luciferase assays, PC12-NFAT-Luc cells were plated at 40,000 cells/100 μl/well in a 96-well plate. The following day, the culture medium was completely removed and replaced with medium containing the agents to be tested in the experiment. Antagonists or inhibitors were applied 30 min before stimulation with ATP. After 3 h of incubation, the medium was removed, and the cells were lysed in passive lysis buffer (Promega, Madison, WI) by vigorous shaking for 1 min at room temperature. Luciferase activity was determined by mixing an aliquot of the lysate with 4 vol of the luciferase assay mix according to Gaunitz and Papke [64] and measuring light emission in an Orion Microplate Luminometer (Berthold Detection Systems, Pforzheim, Germany). All data were obtained from triplicate wells.

### Western blotting

Whole cell lysates for immunodetection of ERK phosphorylation were prepared by collecting the cells by centrifugation in a micro test tube and subsequent lysis with 100 μl/well (in a 6-well plate) boiling SDS-buffer (20 mM Tris, pH 7.4; 1% SDS). The phosphorylation of ERK1/2 was assessed by Western blot analysis using polyclonal rabbit antibodies specific for phospho-p44/42 MAPK (Erk1/2) (Thr202/Tyr204) and total p44/42 MAPK (Cell Signaling Technology, Danvers, MA). The Western blots were developed using horseradish peroxidase-coupled secondary antibodies and chemiluminescence detection. Signal intensities were quantitated with a LAS-3000 CCD imaging system and the AIDA Image Analyzer 5.0 program (Raytest, Straubenhardt, Germany).

### Quantitative RT-PCR

To quantify the mRNA levels of RCAN1 and BDNF, 3x10^6 ^PC12-NFAT-Luc cells were plated in 60 mm culture dishes. The next day, the medium was changed, and ATP and FK506 were added. The cells were incubated with ATP for 3 h, while FK506 was added 30 min before stimulation with ATP. The RNeasy Mini Kit (Qiagen, Hilden, Germany) was used for RNA purification according to the manual. For the cDNA synthesis, 1 μg of total RNA was reverse transcribed using 1 μg of oligo(dT) and MMLV reverse transcriptase (Promega) at 40°C for 1 h. The resulting cDNAs were analysed using a LightCycler 480 system and SYBR Green master mix reagent (Roche Applied Science), using the following PCR conditions: 5 min initial denaturation at 95°C, followed by 45 cycles of 10 s at 95°C, 10 s at 50-62°C, 15 s at 72°C and 1 s at 74°C. The sequences of the oligonucleotide primers used for the specific detection of the rat RCAN1-4 transcript [GenBank NM_153724.2] and the exon IV-containing *Bdnf *transcript [GenBank EF125679.1] are given in the Supplementary material (Additional file 1). The beta-2 microglobulin gene was used as a housekeeping gene for normalization.

### Endpoint RT-PCR

The sequences of the primers used for the amplification of the P2X and NFAT sequences are given in the Supplementary material (additional file 1). The REDTaq PCR Reaction Mix (Sigma) was used under the following PCR conditions: 2 min initial denaturation at 94°C, followed by 35 cycles of 30 s at 94°C, 30 s at 52-58°C and 1 min at 72°C. The positive control plasmid for amplification of P2X7 cDNA was kindly provided by Günther Schmalzing in our Institute.

### Statistical analysis

The GraphPad Prism 5.0 program (GraphPad Software, La Jolla, CA) was used for curve fitting by nonlinear regression and statistical analysis. Results were tested for statistical significance by ANOVA and Bonferroni's correction for multiple comparisons.

## Abbreviations

α,β-MeATP: α,β-methylene ATP; BDNF: brain-derived neurotrophic factor; BzATP: 4-benzoylbenzoyl ATP; NFAT: nuclear factor of activated T cells; PMA: phorbol 12-myristate 13-acetate; PPADS: pyridoxal-phosphate-6-azophenyl-2',4'-disulfonate; RCAN: regulator of calcineurin; RT-PCR: reverse transcription polymerase chain reaction; SOCE: store-operated Ca^2+ ^entry.

## Competing interests

The authors declare that they have no competing interests.

## Authors' contributions

PP performed the most of the luciferase experiments and the Western blots. GS planned and carried out all RT-PCR experiments and the luciferase assays shown in Figure 4B and participated in the writing of the manuscript. WB designed and coordinated the study and wrote the manuscript. All authors read and approved the final manuscript.

## Supplementary Material

Additional file 1 **Primer sequences**. This PDF file lists the oligonucleotide sequences of the PCR primers used in this study. Click here for file
