# Supplementary material for: Extracellular ATP activates NFAT-dependent gene expression in neuronal PC12 cells via P2X receptors
Source: BMC Neurosci. 2011 Sep 23;12:90. doi: 10.1186/1471-2202-12-90 (PMC3189881; doi:10.1186/1471-2202-12-90)
Supplement: Additional file 1 — Primer sequences. This PDF file lists the oligonucleotide sequences of the PCR primers used in this study. [file 1471-2202-12-90-S1.PDF]

Additional file 1: Sequences of oligonucleotide primers

Oligonucleotide primers used for detection of NFAT isoforms (according to Ref. 34)

primer	Sequence 5´ → 3´	product size (bp)
NFATc1for	AGATGGTGCTGTCTGGCCATAACT	156
NFATc1rev	TGCGGAAAGGTGGTATCTCAACCA	
NFATc2for	TCACAGCTGAGTCCAAGGTTGTGT	106
NFATc2rev	AGCATGTTAGGCTGGCTCTTGTCT	
NFATc3for	TGGCATCAACAGTATGGACCTGGA	147
NFATc3rev	TTTACCACAAGGAGAAGTGGGCCT	
NFATc4for	ATCACTGGCAAGATGGTGGCTACA	128
NFATc4rev	AGCTTCAGGATTCCAGCACAGTCA	

Oligonucleotide primers used for detection of P2X isoforms

Oligonucleotides for P2X1-3 and P2X4for were kindly provided by Ralf Hausmann (Institut of Pharmacology, RWTH Aachen University) and match the murine transcripts. Primers for the detection of P2X5-7 were designed according to Ref. 44.

primer	Sequence 5´ → 3´	product size (bp)
mP2X1	ATGTTCTCCTGCAGGCCCAG	296
	GTGCAGAATGGGACAAACCG	
mP2X2	ACAGGAGGCCTTTGTCCCATATGC	251 and 461[a]
	CACCACCACCACTCGAACTCTCATC	
mP2X3	GGCCTGGTCACTGGTGAACA	285
	AAGATGGAGAATGGCAGCGA	
mP2X4	CCGGAAAGACCCTGCTCGTA	251
	AGGGACCTGGCCGGCAAAGA	
rP2X5	GCCGAAAGCTTCACCATTTCCATAA	418
	CTATCACATCAAAGCGGATGCCGTAGG	
rP2X6	AAAGACTGGTCAGTGTGTGGCGTTC	520
	TGCCTGCCCAGTGACAAGAATGTCAA	
rP2X7	GTGCCATTCTGACCAGGGTTGTATAAA	354
	GCCACCTCTGTAAAGTTCTCTCCGATT	

[a] alternative splicing variants (P2X2a [GenBank NM_053656.2] and P2X2b [GenBank Y10473]). The identification of the PCR products was confirmed by sequencing.

Oligonucleotide primers used for qRT-PCR. The primers sequences for rat RCAN1-4 were taken from Lee et al. 2010 (*Hum Mol Genet* 19:468-479).

RCAN1-4	GCCCGTTGAAAAAGCAGAAT 191	191
	GACAGGGGGTTGCTGAAGTT	
rmBDNF4	GCGTGCGAGTATTACCTCCGCC	245
	GGTCATCACTCTTCTCACCTGGTGG	
beta2M	CCGTGATCTTTCTGGTGCTTGTCT	272
	ATCGGTCTCGGTGGGTGTGAAT	
